# Ultra Low Dose CT Pulmonary Angiography with Iterative Reconstruction

**DOI:** 10.1371/journal.pone.0162716

**Published:** 2016-09-09

**Authors:** Andreas Sauter, Thomas Koehler, Alexander A. Fingerle, Bernhard Brendel, Vivien Richter, Michael Rasper, Ernst J. Rummeny, Peter B. Noël, Daniela Münzel

**Affiliations:** 1 Department of diagnostic and interventional Radiology, Technische Universität München, Munich, Germany; 2 Philips GmbH, Innovative Technologies, Research Laboratories, Hamburg, Germany; 3 Department of diagnostic and interventional Radiology, Universitätsklinikum Tübingen, Tübingen, Germany; 4 Lehrstuhl für Biomedizinische Physik, Physik-Department & Institut für Medizintechnik, Technische Universität München, Garching, Germany; Universidad Francisco de Vitoria, SPAIN

## Abstract

**Objective:**

Evaluation of a new iterative reconstruction algorithm (IMR) for detection/rule-out of pulmonary embolism (PE) in ultra-low dose computed tomography pulmonary angiography (CTPA).

**Methods:**

Lower dose CT data sets were simulated based on CTPA examinations of 16 patients with pulmonary embolism (PE) with dose levels (DL) of 50%, 25%, 12.5%, 6.3% or 3.1% of the original tube current setting. Original CT data sets and simulated low-dose data sets were reconstructed with three reconstruction algorithms: the standard reconstruction algorithm “filtered back projection” (FBP), the first generation iterative reconstruction algorithm iDose and the next generation iterative reconstruction algorithm “Iterative Model Reconstruction” (IMR). In total, 288 CTPA data sets (16 patients, 6 tube current levels, 3 different algorithms) were evaluated by two blinded radiologists regarding image quality, diagnostic confidence, detectability of PE and contrast-to-noise ratio (CNR).

**Results:**

iDose and IMR showed better detectability of PE than FBP. With IMR, sensitivity for detection of PE was 100% down to a dose level of 12.5%. iDose and IMR showed superiority to FBP regarding all characteristics of subjective (diagnostic confidence in detection of PE, image quality, image noise, artefacts) and objective image quality. The minimum DL providing acceptable diagnostic performance was 12.5% (= 0.45 mSv) for IMR, 25% (= 0.89 mSv) for iDose and 100% (= 3.57 mSv) for FBP. CNR was significantly (p < 0.001) improved by IMR compared to FBP and iDose at all dose levels.

**Conclusion:**

By using IMR for detection of PE, dose reduction for CTPA of up to 75% is possible while maintaining full diagnostic confidence. This would result in a mean effective dose of approximately 0.9 mSv for CTPA.

## Introduction

Computed tomography pulmonary angiography (CTPA) is the most commonly used imaging modality to confirm/rule-out suspected pulmonary embolism (PE) [1–3]. CTPA is available at all times in most hospitals, can be performed quickly and is relatively cost efficient. With improvements in CT technology, high diagnostic accuracy can be achieved with sensitivities up to 92% and specificities up to 95% [4]. However, CTPA involves a high radiation exposure with an estimated dose-average of 10.7 mSv [5, 6]. It must be noted that use of CTPA is limited in pregnant women as well as in patients with chronic kidney failure and iodine allergy. In these patients, indication for CTPA must be considered carefully and CTPA is eventually not possible.

In the last decades the number of CT scans in the United States raised rapidly from approximately 3 million in 1980 to about 62 million in 2006 [7], resulting in increasing collective radiation exposure. Radiation exposure is associated with potentially increased lifetime risk of malignancy and danger of gametal damage, especially when applied to younger or pregnant patients [7, 8]. Ionizing radiation is an established risk factor for breast cancer and there is a substantial radiation exposure to the breast in CTPA [9]. Therefore, dose reduction is particularly needed in younger patients. Various techniques for dose reduction have been developed and partially implemented in daily clinical routine, following the principle “as low as reasonably achievable” (ALARA) [10].

Apart from the use of shielding techniques and reduction of anatomical scan coverage, dose can also be reduced via reducing tube peak kilo voltage (kVp) or tube current (mA) and by the use of new methods such as z-axis modulation [11, 12]. Multiple studies proved that suitable image quality of CTPA can be achieved even when tube voltage is reduced to 100 kVp or even 80 kVp, resulting in significantly lower radiation exposure [13]. However, reduced tube voltage leads to higher image noise, especially in obese patients. To compensate for image noise, higher tube currents are needed, therefore in total only limited reduction of radiation dose is achieved [14–16].

In order to decrease radiation dose even further, additional reduction of tube current must be aspired. Depending on the individual patient’s constitution, different tube currents are needed to obtain suitable image quality. Highly reduced tube current can lead to impaired image quality and therefore missed diagnoses become more likely [17–19].

Advances in processing power and development of new and faster algorithms have enabled a wide-spread clinical use of iterative reconstruction methods. These iterative reconstruction algorithms make improvements in image quality and in consequence reduction of radiation dose possible, even in obese patients [20–25]. Iterative reconstruction has been introduced into clinical routine during the last 5 years. First generation iterative algorithms have already led to a significant reduction of image noise in comparison to FBP [23, 26].

In this study a new iterative algorithm was compared to a first-generation iterative algorithm (iDose) and the standard filtered back projection (FBP) at different simulated tube current levels.

The objective was to evaluate the diagnostic performance of a new generation iterative reconstruction algorithm (IMR) in low and ultra-low dose CTPA with focus on image quality and diagnostic confidence in diagnosing PE.

## Materials and Methods

### Patient population

This single centre study was approved by the institutional review board (Klinikum rechts der Isar, Technische Universität München) and written informed consent was obtained from all patients before enrolment.

16 patients with PE (7 male, 9 female) were included in this study. PE was subclassified as central, segmental, or subsegmental. Multiple localizations were also possible. No preselection regarding patient weight, age, sex or other characteristics was performed. Image quality can be influenced by other criteria (e.g. unfavourable contrast phase, motion artefacts). These images are not sufficient for diagnostic uses, regardless of the reconstruction algorithm. As consequence, only examinations with suitable conditions were used for this study.

### CTPA image acquisition

All patients were examined using a 256-slice multidetector CT (Brilliance iCT; Philips Healthcare, Cleveland, OH, USA) using a standard CTPA protocol which involves a body mass index (BMI) adjustment of the tube current. Patients were placed in supine position on the scanner couch. After an anteroposterior and lateral scout to define the optimal scan region, 60 ml of contrast agent (Iomeron 400 MCT, Bracco Imaging Deutschland GmbH, Konstanz, Germany) were injected intravenously with an injection rate of 3.5 ml/s using a dual syringe injection system (Stellant, MEDRAD, Inc., Indianola, Pennsylvania). The bolus tracker was placed within a region-of-interest (ROI) in the pulmonary trunk and was used to ensure optimal contrast enhancement (threshold for scan start: 100 HU). The scan was performed craniocaudally with a pitch of 0.9 and a 128x0.625-mm detector configuration. Tube voltage settings depended on BMI with 120 kVp for a BMI >25 kg/m^2^ and 100 kVp for a BMI <25 kg/m^2^. Mean tube current was 106 mA (range 52–240 mA, depending on patient’s BMI), resulting in a mean dose-length-product (DLP) of 247 mGy*cm (range 74–695 mGy*cm) with a mean effective dose of 3.6 mSv (range 1.1–10.1 mSv).

### Simulation of low-tube-current images and reconstruction

Until recently, to compare different dose levels in clinical images, multiple CT scans had to be performed, resulting in high radiation exposures. However, with the lately implemented low-dose simulation tool, it became possible to simulate lower dose data retrospectively from preexisting clinical routine data as described by Muenzel et al. and Zabic et al. [27, 28]. CTPA raw data were used to simulate CTPA scans with reduced tube currents, resulting in dose levels (DL) of 50%, 25%, 12.5%, 6.3% and 3.1% percent of the original dose. All other imaging parameters remained identical. All obtained CTPA images (tube current levels of 100%–3.1%) were reconstructed with FBP, iDose, and IMR, resulting in a total of 288 datasets (6 different tube current levels, 3 different algorithms, 16 patients). All data sets were reconstructed in axial view with slice thickness of 3 mm, a 512 image matrix and 350 mm field of view.

### Subjective image analysis and diagnostic confidence

Each data set was independently evaluated by two blinded radiologists (3 and 4 years of clinical experience in CT diagnostics) regarding the following criteria: image noise (5 levels: 1 = minimal/no image noise, 2 = less than average noise, 3 = average noise, 4 = above average noise, 5 = unacceptable image noise); classic artefacts (4 levels: 1 = no artefacts, 2 = minor artefacts not affecting the diagnostic decision making, 3 = major artefacts affecting visualization of structures, diagnosis still possible, 4 = substantial artefacts making the image non diagnostic); ring artefacts (yes/no), image quality (4 levels: 1 = unacceptable for diagnostic purposes, 2 = somewhat suboptimal, 3 = good, 4 = excellent); diagnostic confidence in detection of central and peripheral PE as well as overall diagnostic confidence (4 levels: 1 = poor confidence, 2 = confident only for limited clinical situation, 3 = probably confident, 4 = completely confident); artificial image appearance (4 levels: 1 = none, 2 = week, 3 = moderate, 4 = strong). All image analysis was performed on monitors approved for diagnostic use (Totoku MS 2512/3215).

Raters were asked to evaluate central, segmental and subsegmental pulmonary arteries in each data set as positive or negative for PE. PE in multiple localizations were possible. As there is no gold standard for the detection of PE, the clinical report as well as a re-evaluation of the clinical available images by the author were used to determine the correctness of the ratings.

### Objective image quality

In order to measure image quality objectively, contrast-to-noise ratio (CNR) for the main pulmonary artery as a central vessel (central contrast to noise radio—cCNR) and contrast to noise ratio for a segmental artery as a peripheral vessel (peripheral contrast to noise ratio—pCNR) was obtained. For this cause, a circular ROI was placed in the main pulmonary artery to acquire the corresponding Hounsfield units (HU) of a central vessel (HU_CV_), of a paraspinal muscle (HU_M_) and a segmental artery as a peripheral vessel (HU_PV_). We obtained attenuation and standard deviation (SD) of all ROIs. SD in the central and peripheral vessel was defined as noise. cCNR was calculated as [HU_CV_—HU_M_]/noise and pCNR as [HU_PV_—HU_M_]/noise.

### Statistical analysis

Continuous data is expressed as arithmetic mean ± SD. A two-tailed student’s t-test was performed for comparison of cCNR and pCNR values, respectively. Results of subjective image quality assessment are expressed as medians and shown as box-and-whisker plots. They were analyzed using Wilcoxon signed-rank test. A p-value ≤ 0.05 was considered statistically significant. All statistic testing were computed with IBM SPSS Statistics 23 and 24.

## Results

### Subjective image analysis

For images reconstructed with iDose and IMR, median subjective image quality was higher than for images reconstructed with FBP at all dose levels from 100% to 12.5%. At DL of 3.1%, median image quality was 1 (unacceptable for diagnostic purposes) for all algorithms. Median image quality was higher after reconstruction with IMR than after reconstruction with iDose at DL of 6.3% and 25% (median of 1.5 vs. 1 and 3 vs. 2.5, respectively).

Median subjective image noise was lower in images reconstructed with IMR and iDose compared to images reconstructed with FBP.

Median subjective image noise in IMR-images was lower than in iDose-images at all dose levels with the exception of the 100% DL, where both iDose and IMR showed a median subjective image noise of 2 (less than average noise). IMR-images presented a median of 2 (less than average noise) at all DLs of 100–12.5%. Even at 3.1% and 6.3% DL, IMR-images had a median subjective noise level of 3 (average noise). For iDose-images, subjective image noise was higher at lower dose levels (2 at 100%, 2.5 at 50%, 3 at 25% and 12.5% and 4 at 6.3% and 3.1%). FBP-images showed a median of 3 (average noise) at 100% DL and a median of 4 (unacceptable image noise) at all other dose levels (3.1%–50%).

In IMR and iDose-images, the raters detected fewer artefacts compared to FBP-images at all dose levels without significant difference between iDose and IMR images at any dose level (e.g. at 12.5% DL median of 2 for IMR, 3 for iDose and 4 for FBP).

At all dose levels, median artificial image appearance after reconstruction with IMR was higher than after reconstruction with iDose or FBP.

### Diagnostic confidence in detection of PE

**Fig 1:**
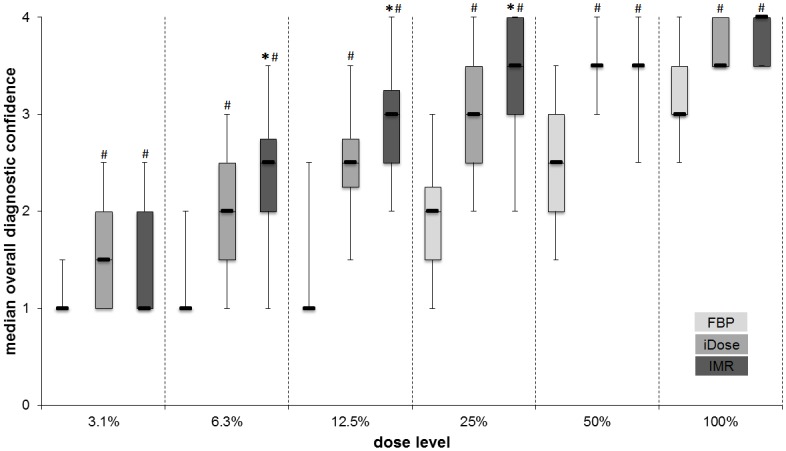
Overall diagnostic confidence. Medians of overall diagnostic confidence in detection of PE shown as box-and-whisker plots at different dose levels (3.1–100% of the original dose level). IMR performs significantly better, providing high diagnostic confidence also at 25%, 12.5% and 6.3% of the original radiation dose. * = significance compared to iDose at the corresponding dose level (* = p<0.05, ** = p<0.01). # = significance compared to FBP at the corresponding dose level (p<0.01). Values are shown as the mean of the medians of both raters. *FBP = filtered back projection*, *iDose = iterative dose reduction*, *IMR = iterative model reconstruction*.

Diagnostic confidence in detection of PE was evaluated for central PE and for peripheral PE. In addition, overall diagnostic confidence in detection of PE was evaluated (Fig 1).

By using iDose and IMR reconstruction, significantly (p < 0.01) higher diagnostic confidence could be obtained than by using FBP reconstruction. This finding is applicable to all divisions of PE (central, peripheral, overall) and to all dose levels.

The median diagnostic confidence in detection of PE in IMR-images was 3 (probably confident) or higher at all dose levels from 12.5%–100%. For iDose-images, a median of 3 or higher was obtained only for dose levels from 25%–100%.

At lower dose levels, IMR-images produced a higher median diagnostic confidence (higher median in IMR-images than in iDose-images at 12.5% DL for all divisions of PE and at 6.3% DL for central and peripheral PE).

At the lowest dose level of 3.1%, medians for diagnostic confidence in detection of PE for all reconstruction algorithms were 1.5 or 1 (poor confidence).

In IMR-images the median regarding diagnostic confidence in detection of PE was 4 (completely confident) for central PE at DL of 25%, 50% and 100%.

The median level of diagnostic confidence was almost similar in images reconstructed with IMR at 50% DL and with iDose at 100% DL for all divisions.

Comparison of diagnostic confidence in detection of central PE for IMR-images at 25% DL and iDose-images at 100% DL showed no significant difference.

At high dose levels (100% and 50%) there was no significant difference between iDose and IMR images. However, at lower dose levels (25% and 12.5%), IMR-images provided a higher median diagnostic confidence in detection of PE for all division than iDose-images.

Images reconstructed with IMR produced very high levels of diagnostic confidence in detection of PE in all divisions from 100%–25% DL (median of 3 or higher), without significant differences in diagnostic confidence between 50% and 25% DL.

### Detection of PE

On the basis of the full-dose images, no PE (central, segmental, and subsegmental) was missed by either rater in IMR-reconstructed images at dose levels of 12.5%-100%, resulting in a sensitivity of 100%. Same applies for iDose at dose levels of 25%-100%. With FBP reconstruction, only at 100% DL sensitivity was 100% for central, segmental and subsegmental PE. By using FBP-reconstruction at 12.5% DL, sensitivity was 35% for central PE, 23% for segmental and 19% for subsegmental PE.

With IMR and iDose reconstruction, there was a sensitivity of 58% for central PE at the lowest DL (3.1%), whereas with FPB-reconstruction sensitivity was only 6%.

### Objective image quality

**Fig 2:**
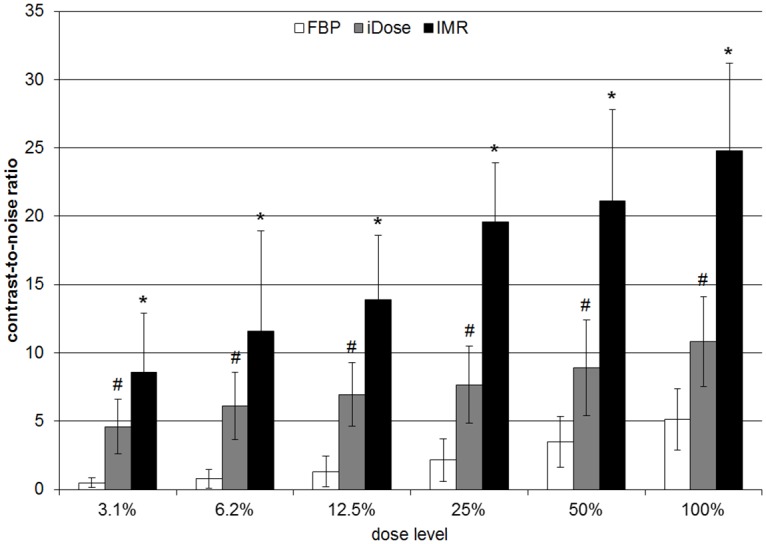
Contrast-to-noise ratio. Contrast-to-noise ratio of a central pulmonary artery as a central vessal (cCNR) for all reconstruction algorithms and dose levels shown as mean ± standard deviation. * = significance compared to FBP and iDose at the corresponding dose level (p < 0.001). # = significance compared to FBP at the corresponding dose level (p < 0.001). Significance levels were calculated using Student's t-test. *FBP = filtered back projection*, *iDose = iterative dose reduction*, *IMR = iterative model reconstruction*.

At all dose levels, cCNR (Fig 2) and pCNR were significantly (p < 0.01) higher for IMR-images compared to iDose and FBP-images (except pCNR for IMR vs. iDose at 6.3% DL, here p = 0.011). iDose-images showed higher cCNR and pCNR than FBP-images (p < 0.001).

In addition, cCNR for IMR-images at DL of 50%, 25% and 12.5% was significantly (p < 0.05) better than for iDose-images at 100% DL.

## Discussion

**Fig 3:**
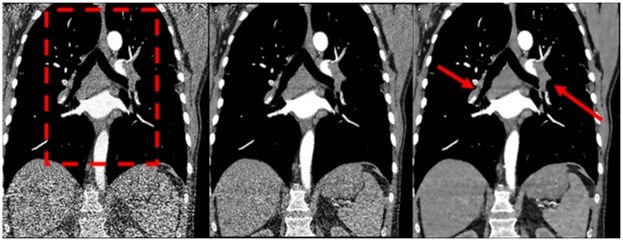
Comparison of FBP, iDose and IMR—coronal view at full dose. Coronal tomographic slices of a 72-year-old male patient. The images were reconstructed with FBP, iDose and IMR (from left to right) at full dose (100% dose-level, meaning 85 mA, 100 kV and 2.25 mSv for this patient). Central and segmental pulmonary emboli can be clearly identified (arrows). The red dashed rectangle indicates the enlarged view in Fig 4. *FBP = filtered back projection*, *iDose = iterative dose reduction*, *IMR = iterative model reconstruction*

**Fig 4:**
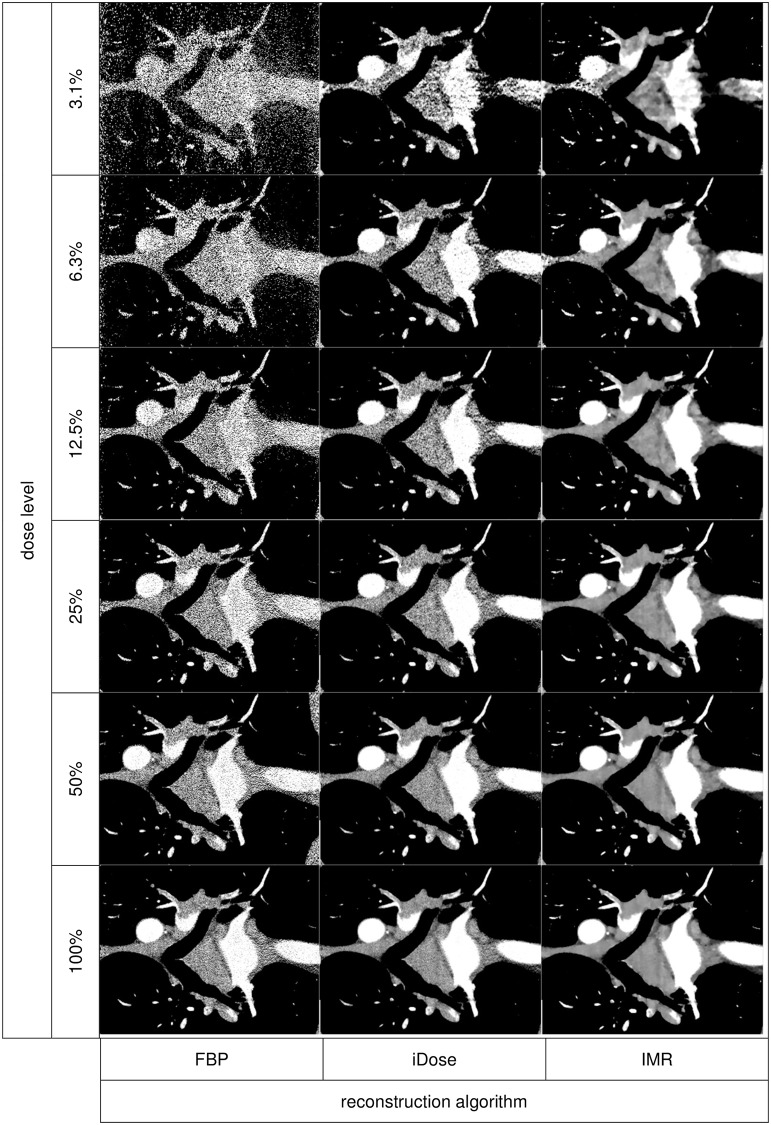
Comparison of FBP, iDose and IMR at dose levels of 3.1–100%. Enlarged view of the coronal tomographic slices of the chest from Fig 3, indicated by the red dashed rectangle. Comparison of FBP (top), iDose (middle) and IMR (bottom) at different dose levels (from left to right: 100%–50%–25%–12.5%–6.3%–3.1%). Emboli are located in the right pulmonary artery, the left upper and lower lobar artery and in several segmental arteries of the left lung. Note the good detectability even at ultra-low dose levels with IMR. With iDose emboli are also detectable but image quality is inferior to IMR. With FBP, emboli are not certainly detectable at lower dose levels. *FBP = filtered back projection*, *iDose = iterative dose reduction*, *IMR = iterative model reconstruction*.

Our study showed that IMR, a next generation iterative reconstruction algorithm, provides excellent diagnostic confidence in the detection of PE even in ultra-low-dose images with a simulated mean effective dose of only 0.9 mSv (Figs 3 and 4).

Due to the potential risk of malignancy or gametal damage caused by exposure to ionizing radiation, reduction of radiation dose is essential [29]. However, a sufficient image quality for diagnosis of PE must be obtained. Using new iterative reconstruction algorithms, significant dose reduction via reduced tube-current and tube-voltage was possible in the last years [13, 26]. However, an even further reduction of radiation exposure must be pursued, as CTPA is frequently used in the clinical routine. As a reduction of tube current below 80 kVp is not reasonable due to the k-edge of iodine, further reduction of radiation dose has to be obtained by reducing tube current [22, 30].

To our knowledge so far no systematic study has examined diagnostic confidence in detection of PE and image quality in simulated low-tube-current CTPA. Multiple CTPA scans with different tube-currents of the same patients are unacceptable due to ethical reasons regarding radiation exposure and the amount of intravenous contrast agent. With recently developed CT system simulation tools, computed simulation of low-tube-current CTPA by the mathematical addition of noise became possible and provides realistic and reproducible images [27].

Five different levels of reduced tube currents were simulated. These simulated low-tube-current images and the corresponding full dose images were reconstructed with three different algorithms: standard FBP, iDose (a first generation iterative reconstruction algorithm), and IMR (a next generation iterative reconstruction algorithm).

Since introducing the iterative reconstruction algorithm iDose in our department, the mean effective dose for CTPA could be reduced by about 34% from 9.7 mSv to 6.4 mSv [26]. In addition, high contrast examinations such as CTPA offer the possibility to lower radiation dose by reduction of tube voltage to 100 kVp in patients with a normal body weight (BMI <25kg/m^2^) [26]. In this study, CTPA scans were performed with a mean effective dose of 3.6 mSv, which is only one third of the average effective dose for CTPA (10.7 mSv) as a recently published study showed [6].

Previous studies proved that images reconstructed with iDose present much better image quality than images reconstructed with FBP, making lower tube currents with a maintained high diagnostic confidence possible [22]. This study confirms these results regarding iDose-images with focus on subjective and objective image criteria.

For FBP, image quality can be examined using objective mathematical methods such as image noise and CNR. However, for images reconstructed with iterative algorithms, mathematical methods are only of impaired relevance concerning classification of image quality. For example, using a strong iterative reconstruction algorithm, image noise could be eliminated completely but blurring would be increased subsequently resulting in limited or missing diagnostic quality. In order to determine if the reconstruction algorithm provides maximum diagnostic quality for a specific indication, subjective image assessment is vital. But, as objective image analysis (CNR and image noise) can be performed in a standardized and repeatable manner, it is an important additional tool when evaluating image quality. Thus objective and subjective image quality assessment is required for evaluation of overall diagnostic quality [28].

Images reconstructed with IMR tend to have an unusual image appearance, which was confirmed by this study. Images reconstructed with iDose and FBP were not classified as having an artificial image appearance, consistent with several past studies which showed that iDose can preserve a natural image appearance [31]. Artificial image appearance is a well-known feature of more recent iterative reconstruction techniques and several authors revealed artificial image appearance as a major setback of diagnostic quality [31, 32]. However, in this study, only a slightly artificial appearance was noted in IMR images. The overall image appearance and the level of diagnostic confidence in detection of PE were not noticeably influenced.

At higher dose levels (50% and 100%), there were little or no differences regarding diagnostic confidence in the detection of PE and most other criteria of image quality between IMR and iDose-images. This is probably due to the already very high image quality after reconstruction with iDose at these dose levels. In low dose and ultra-low dose images, IMR-images showed better results for diagnostic confidence and image quality. However, this superiority of IMR couldn’t be proven significant. One potential reason for the lack of greater superiority of IMR compared to iDose could be the modality as CTPA is a high contrast protocol. In high contrast protocols, even simple iterative reconstruction algorithms (such as iDose) offer a significant improvement of image quality compared to FBP, as the difference in attenuation between the contrast filled lumen and the non-enhanced embolus provide an excellent setting for noise reducing algorithms. In addition, we included only 16 patients in this study, so that tendencies of improved quality parameters may not gain statistical significance.

Compared to standard of care, we found a sensitivity of 100% for central, segmental and subsegmatal PE in images reconstructed with iDose for dose levels of 100–25% and in images reconstructed with IMR for any dose level down to 12.5%. Consequently, dose reduction to a mean effective dose of 0.45 mSv (range 0.13–1.26) without missing any PE was possible.

IMR-images produced similar or better results regarding subjective and objective image quality compared to iDose-images. According to the principle of ALARA, dose should not be lowered to the lowest possible value but only to a value where a very high diagnostic confidence is given. In this study IMR showed the highest diagnostic confidence level in detection of PE. Mean overall diagnostic confidence, as well as diagnostic confidence in detection of peripheral PE was rated as probably confident or higher for 100%, 50% and 25% DL. For central PE, the mean diagnostic confidence was rated completely confident at these dose levels. Regarding these results, a dose reduction of 50% or even 75% (mean effective dose of 1.8 mSv or 0.9 mSv) based on the already low dose used in our department is possible with very little loss of image quality and a very high level of diagnostic confidence.

One possible limitation of the present study is the small number of patients included. Despite examining this relatively small group, significant differences between dose levels and reconstruction algorithms regarding different parameters of image quality were determined. In order to assure that no case of PE is missed at lower dose levels and to prove that IMR is superior to iDose in image reconstruction, a greater number of CTPAs should be simulated with low and ultra-low-tube-current. If future studies would confirm that no case of PE is missed at lower dose levels (e.g. dose level of 50%) and that image quality is sufficient for diagnosis/rule-out of PE, lower tube currents could be established in the day-to-day routine.

Second, in the present study images for radiologists were only available in 3 mm axial slices. In the clinical routine, additional reformations such as a coronal view and axial images with slice thickness of 0.625 mm are available if needed as these additional information can be helpful to detect PE or to clarify questionable findings. However, these additional reformations are essentially required only in rare cases for detection of PE. As the reformations given to the radiologist raters in the present study were identical for all cases, no individual disadvantages could arise from the missing reformations. Therefore, providing axial 3 mm images is sufficient for the purposes of research.

Third, in our study a 256-slice CT scanner was used, which is not available in all departments and results of this study can not necessarily be transformed to other CT scanners.

In conclusion, IMR, a next generation iterative reconstruction algorithm, enables distinct reduction of the individual patient’s radiation dose in CTPA examinations, providing suitable image quality even in sub-mSv images.

## Supporting Information

S1 Table Comparison of FBP, iDose and IMR regarding subjective image criteria. (XLS)

S2 Table Comparison of FBP, iDose and IMR regarding objective image criteria. (XLS)
